# Solvent influence on molecular interactions in the bulk of fluorene copolymer films†

**DOI:** 10.1039/d0ra02058c

**Published:** 2020-06-01

**Authors:** Karina da Silva Dias, Ranylson Marcello Leal Savedra, Carlos Eduardo Tavares de Magalhães, Melissa Fabíola Siqueira

**Affiliations:** Department of Physics, MolSMat – Molecular Simulation of Materials, Laboratory of Computational Simulation (LabSimCo), Federal University of Ouro Preto 35400-000 Ouro Preto MG Brazil melissa@ufop.edu.br; Department of Physics, Laboratory of Polymers and Electronic Properties of Materials (LAPPEM), Federal University of Ouro Preto Ouro Preto MG Brazil

## Abstract

The effect of intermolecular interactions between the chains of the amorphous PFO–MEH-PPV films built from toluene and tetrahydrofuran (THF) were studied by atomistic molecular dynamics simulations, applying a successive solvent removal procedure. In the good solvent toluene, the incidence of topological entanglements is more significant. While in the poor solvent, coplanar interactions between neighbouring segments of the chains were also found, which is characteristics of cohesional entanglements. Structure factor curves of the films showed three peaks associated with the microstructure of the film, as previously reported by WAX diffractogram measurements. Moreover, the good solvent promotes more flexibility in dihedral angles, and the chains become nearer to each other.

## Abstract 2

The effect of intermolecular interactions between the chains of the amorphous PFO–MEH-PPV films built from toluene and tetrahydrofuran (THF) were studied by atomistic molecular dynamics simulations, applying a successive solvent removal procedure.

## Graphical abstract

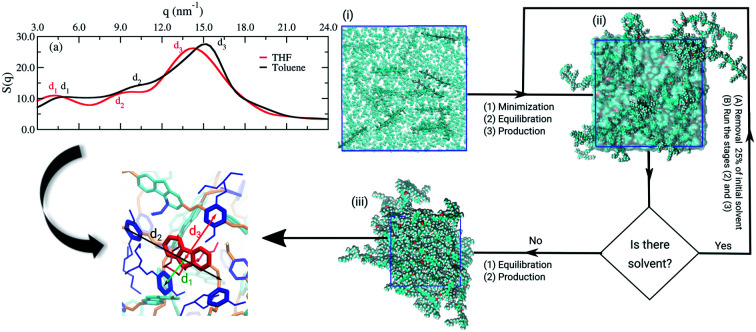

## 1 Introduction

The versatile applications of π-conjugated organic semiconductors for photovoltaic solar cells, light-emitting diodes, field-effect transistors, and other optoelectronic devices, have attracted technological as well as scientific interests. In their potential commercial uses, researchers have been seeking flexible, wearable, and eco-friendly materials.^1,2^

It is well-known that the optoelectronic performance is strongly impacted by the type of aggregation of the chains, which is related to the backbone configurational arrangements, or packing, in solid films.^3–7^ The insertion of side chains in polymers, generally, has the primary purpose of increasing the solubility and processability of these materials.^8–10^ As a consequence, torsions in the polymer backbone are more frequent due to steric factors. Hence, controlling the polymer chains configuration is fundamental to achieving high-performance devices.

The processing parameters chosen for the film production, in particular the solvent, are essential for the control of its photophysical properties, the charge transport, as well as the morphological arrangements of these materials.^3,11–13^ The choice of the solvent for the manufacturing of the polymers has conventionally been made by means of a selection solubility scale.^14–16^ However, producing a highly ordered polymer film is not an easy task due to the nature of the solvent–polymer interactions.^4,15,16^

Fluorene-based polymers (PFO) are attractive conjugated materials for optoelectronic devices because they allow chemical modification to exhibit a broad range in the visible spectrum, high photoluminescence efficiency and good thermal stability.^12,13,17–22^ It is known polyfluorenes adopt two phases, a disordered “glassy” phase with angles between the rings randomly distributed, and the more ordered β-phase (coplanar conformations).^19,23,24^ In general, β-phase and aggregation have been found in poorer solvents and higher molecular weight polymers. The presence of interchain interaction was reported in the β-phase due to the close contact between the aromatic rings.^12,25^ Photophysical studies have shown the photoluminescence quantum efficiency (PLQE) is strongly dependent on local molecular order and conformation of the chains.^19,24,26^ The length of side chains and the concentration of the solution are also important to control the aggregate formation.^27^ Moreover, the flexibility of the backbone chains increases the possibility of intermolecular electron transition occurring, according to Mulliken’s transition moment theory.^28–31^ The PFO–MEH-PPV copolymer has been used as a green colour-conversion material.^12,32^ PFO derivatives with lower molecular weight showed a rod-like conformation enabling π–π stacking structures in its microstructure, while the conformations change to a flexible-coil in higher molecular weight polymers.^32^

To study non-crystalline systems is a complex task since they have no structural organisation pattern. However, molecular simulations have provided valuable contributions to the understanding of organic semiconducting materials, concerning their electronic and structural properties.^28,33–39^ Molecular interactions of a few angstroms govern the microstructural properties, and the most appropriate tool to estimate its characteristics is molecular simulations at the atomistic level. In order to obtain a comprehensive physical understanding, at the molecular level, of the chains arrangements in the bulk of PFO–MEH-PPV films, atomistic molecular dynamic (MD) simulations were employed. The structural behaviours of the backbone chains in films built from tetrahydrofuran and toluene have been compared. The structure factors demonstrate that solvent nature influences the bulk of film packing. The results showed that the incidence of chain segments aligned in parallel to each other was higher in the poorer solvent. This fundamental study provides insights into the correlation between chain packing factors with the choice of the solvent in the processing stage of an amorphous polymer film.

## 2 Methodology

**Fig. 1:**
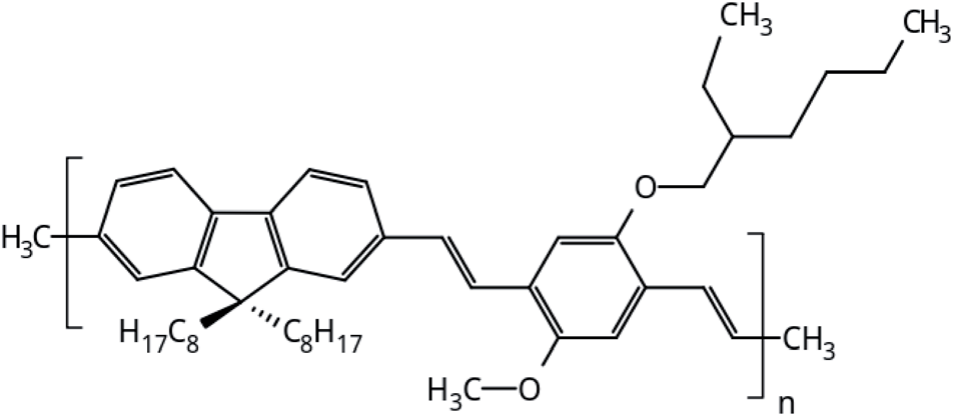
Monomeric unit of PFO–MEH-PPV (poly[(9,9-dioctyl-2,7-divinylene-fluorenylene)-*alt-co*-2-methoxy-5-(2-ethyl-hexyloxy)-1,4-phenylene vinylene), with methyl as the end capped group.

The focus of this work is to present a detailed investigation of the solvent influence in polymer films aggregation. PFO–MEH-PPV is an interesting copolymer for this purpose because its side chains provide more solubility of the polymer and ring twists in the backbone chain (Fig. 1). Thus, the structural properties of PFO–MEH-PPV film models built from tetrahydrofuran (THF) and toluene were evaluated through a gradual solvent removal procedure in atomistic molecular dynamics (MD) simulations.

### 2.1 First principle calculations

The starting structure of PFO–MEH-PPV tetramer used in MD simulations was obtained from full optimisation, using first principle calculations based on Density Functional Theory (DFT). The chosen hybrid functional B3LYP was employed associated with the 6-31G(d,p) Pople’s basis set, implemented in the Orca package, version 2.7.^40–42^

### 2.2 PFO–MEH-PPV films

The initial models of both films were built from a cubic box with a 30.0 nm edge adding randomly 20 tetramers chains of PFO–MEH-PPV, and completing the volume with 7000 solvent molecules, considering periodic boundary conditions. In these simulations the poor solvent THF and the good solvent toluene were used. The first boxes were thermodynamically equilibrated, and later we initiated the steps for the gradual processes of removal of solvent. For that, 25% of the most energetic solvent molecules were excluded from the initial model. This procedure continued until the film formation occurred.

### 2.3 Molecular dynamics simulation details

**Fig. 2:**
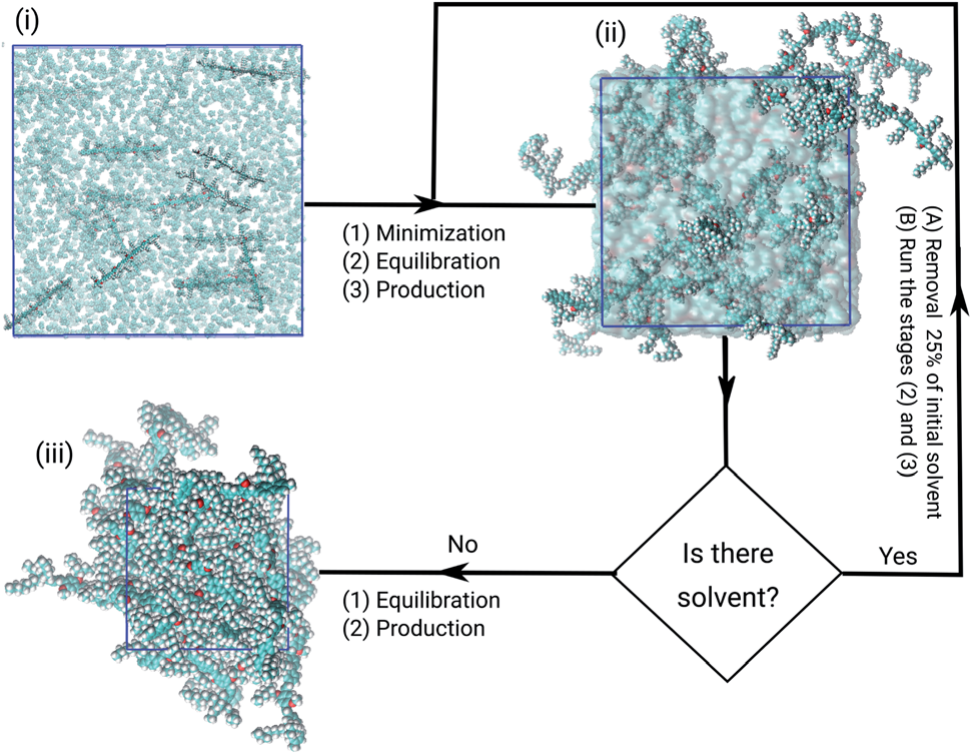
Flowchart summing up the steps of obtaining the models of PFO–MEH-PPV in the solutions until the formation of films. The first state (i) is the initial configuration before energy minimization; the second shows an equilibrated state of the polymer–solvent system (ii) and the bulk of amorphous PFO–MEH-PPV film (iii). In the PFO–MEH-PPV chains the carbons are shown in black, oxygens (red) and hydrogens (white), solvent (cyan surface) and the box edge (blue).

All models were submitted to the same simulation protocols of minimisation, equilibration, and production, as illustrated in Fig. 2. The temperature and pressure were kept constant, at 300 K and 1.0 bar. In the minimisation procedure, the gradient descent method was used to avoid strong repulsive contacts between the atoms of neighbouring molecules. In the equilibration protocol, the NVT and the NPT ensembles were employed to adjust the configurational arrangements in the box.^33^ Each equilibration step was simulated over 10.0 ns with time-step of 1 fs.

After solvent removal, the film production stages were performed over 110 ns. We chose to analyze the final 100 ns of all trajectories afterward the equilibration steps. A canonical velocity rescaling thermostat was applied at 300 K with *τ* = 0.1 ps.^43^ Also, the bond lengths were constrained to their equilibrium values by using the LINCS algorithm.^44,45^ The neighbouring list for the calculation of nonbonded interactions was updated every 10-time steps with a cut-off of 1.0 nm. The same cut-off was used for the Lennard-Jones potential. Moreover, the particle mesh Ewald (PME) method for electrostatic calculations was used.^46,47^ Finally, in the production steps, the Parinello–Rahman barostat was applied isotropically to maintain the system pressure at 1.0 bar, with the time constant of 1.0 ps.^48^

The atomistic molecular dynamics simulations were performed with the GROMACS 2018 package.^49,50^ All molecules were modelled with the GROMOS force field using the parameter set known as 53A6.^51^ The stabilised system models, extracted from the trajectories, were depicted by representative plots produced using the molecular visualisation programme VMD version 1.9.4a12.^52^

## 3 Results and discussion

**Fig. 3:**
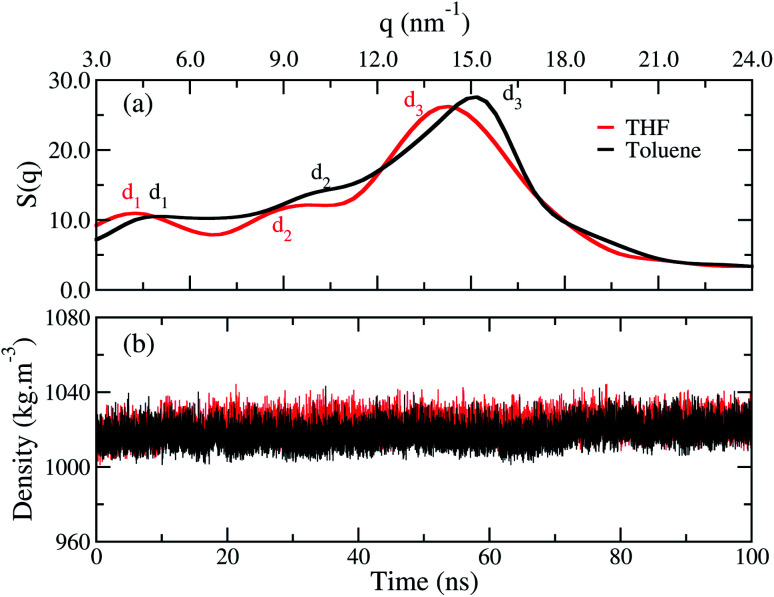
(a) Structure factor and (b) average density calculated over time for the film built from THF (red line) and for the film from toluene (black line).

The structure factor curves, Fig. 3(a), show three characteristic peaks for both films. The curves exhibit a displacement from each other, where the good solvent toluene shifted the peak to higher values of *q*. These results indicate the chains in the bulk of the film adopt different arrangements according to the solvent chosen. In detail, the three main peaks *d*_1_, *d*_2_, and *d*_3_, calculated with *d*_*i*_ = 2π/*q*_*i*_, are listed in Table 1. These peaks are, respectively, associated to the intermolecular distance between the oligomers separated by the side chains, the intramolecular distance between monomer units, and the interplanar π-stacking gap between the aromatic backbone rings. Furthermore, contributions of rings and side chains for the structure factors profile analysed separately, show that independently of solvent the rings and side chains of fluorene make a significant contribution to the *d*_1_ peak. At the same time, *d*_2_ is related to both rings, and *d*_3_ is formed by a majority contribution of all side chains, as shown in Fig. S1, see ESI.† A similar profile was also reported by wide-angle X-ray scattering (WAX) measurements for polymer films obtained from chloroform.^12^ The correspondence between theoretical and experimental results is shown in Table 1, and it is most evident for the film formed from the THF. These results indicate the toluene yields a more compact molecular architecture of the chains in the film than THF.

**Table 1:** Distances associated to the structure factor peaks estimated using MD simulations. The experimental values extracted from literature were measured in CCl_4_ (ref. 12)

System	*d*_1_ (nm)	*d*_2_ (nm)	*d*_3_ (nm)
Film from THF	1.52	0.68	0.44
Film from toluene	1.29	0.63	0.42
Experimental^12^	1.59	0.79	0.45

Though the structure factor has shown that the distances between the chains are smaller in toluene, Fig. 3(b) shows that both films have an equivalent density, independently of the solvent used during their processing. Thus, the difference between the films occurs at the microstructure, and the density has no suitable sensitiveness to distinguish each profile.

**Fig. 4:**
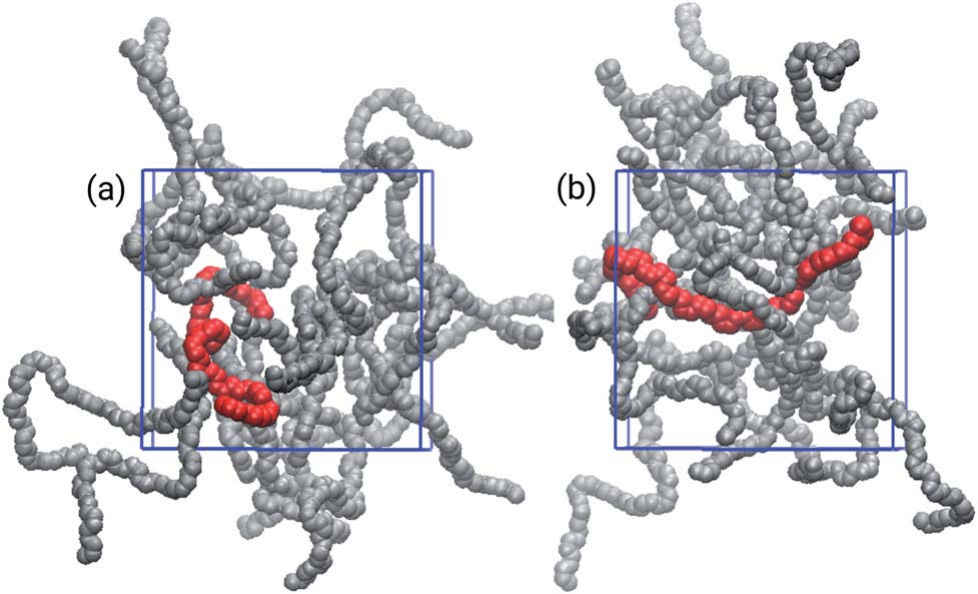
Three-dimensional snapshots of the bulk of film models built from (a) THF, and (b) toluene. The highlighted chains, in red, show typical entanglement configurations: (a) cohesional and (b) topological.

The choice of solvent affected the chain distributions in the film, as depicted in Fig. 4. The chains of the bulk from THF exhibit a significant amount of cohesional entanglements, with local parallel alignments of segments due to van der Waals interactions. On the other hand, with toluene topological entanglements were also found, with backbone chains more elongated and crossing to other neighbouring chains. It had been proposed that locally planar regions of the polymers enable traps for holes because there is evidence the high energy valence orbitals are localized in these planar segments of the polymer.^16,53,54^

**Fig. 5:**
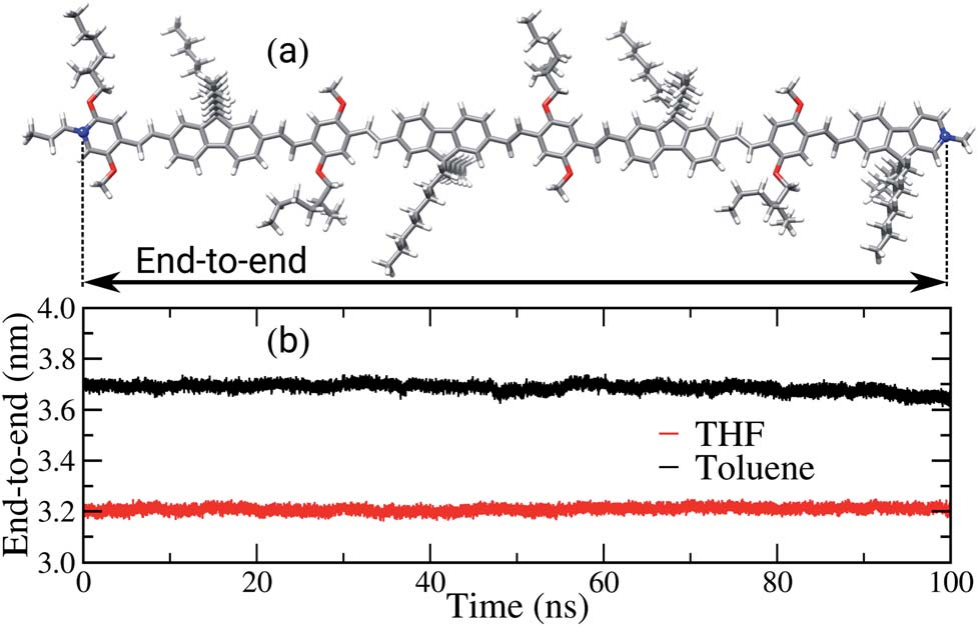
(a) Schematic illustration of end-to-end distance, arrows between the blue atoms and (b) time evolution of the PFO–MEH-PPV end-to-end distance, calculated for the film formed from THF (red) and for toluene (black).

Another property affected by the solvent nature is the end-to-end distance of the backbone chains, as shown in Fig. 5. For the sake of comparison, the length of the backbone chain calculated in the gas phase, using DFT, is approximately 6.5 nm, as illustrated in Fig. 5(a). In the bulk of the films, the average end-to-end distances are 3.7 nm and 3.2 nm for the films built from toluene and THF, respectively. Therefore, in both films a significant decrease of this property in comparison to the initial conformation occurred, *i.e.* to around half of the initial length. The decrease of the chains’ length is expected, considering the driving forces behind interactions between the chains in the amorphous condition of the bulk and the solvent effect during the film formation. However, the poor solvent induces intramolecular folding, shortening the backbone chains, while in the good solvent there is a competition between the interactions solvent–polymer and polymer–polymer, promoting the formation of both cohesional and topological entanglements.

**Fig. 6:**
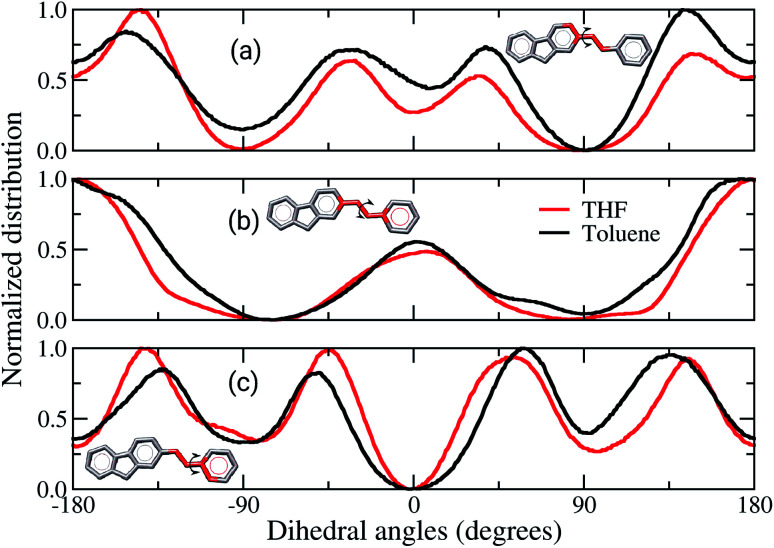
Average of backbone dihedral angles estimated for the films built from THF (red lines) and toluene (black lines), respectively. Three dihedrals were evaluated: (a) fluorene–vinyl, (b) vinyl, and (c) vinyl–MEH-PPV. Side chains were omitted in these representations.

Comparisons between dihedral angle distribution profiles, Fig. 6, show similar behaviours between both solvents, despite the slight quantitative displacement. The dihedral involving the atoms of the rings linked to the vinyl, Fig. 6(a) and (c), are twisted by 30° from a planar configuration. Although these data are the most statistically meaningful, the planar conformations cannot be neglected. It is noticed that the dihedral angles of vinyl groups are mostly planar, Fig. 6(b). Therefore, in brief, these data show that the chains assume preferentially non-planar conformations, hampering the π-stacking interactions in the solid-state. Fig. 6(a) and (b) also show broader curves for the film built from toluene, which demonstrate that the angles between the vinyl and adjacent rings twist more than in film from THF. It indicates the former film has more flexible backbone chains than the latter. Other works have reported similar behaviour for the backbone of amorphous organic semiconductors, suggesting that deviations from planarity are due to Coulomb and van der Waals contributions.^36,37,55^

**Fig. 7:**
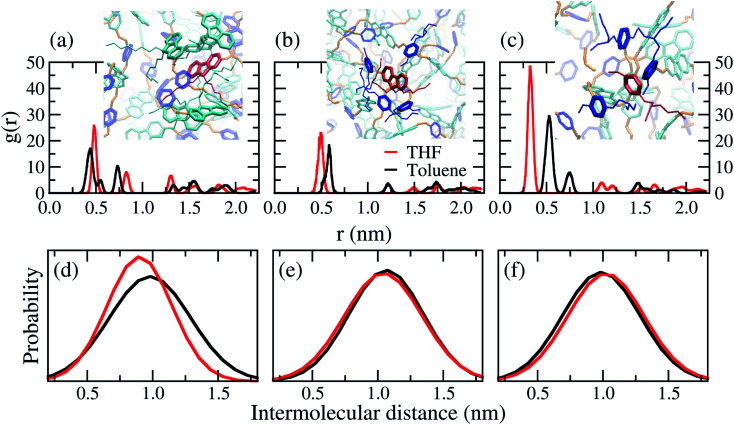
Radial distribution function (RDF) and intermolecular ring distances distribution, estimated between the centres of mass of the rings, for the chains in the bulk of PFO–MEH-PPV. Wall-eye stereogram of a representative configuration for the molecular interactions between (a) FO–FO, (b) FO–MEH-PPV and (c) MEH-PPV–MEH-PPV. The distance between (d) FO–FO, (e) FO–MEH-PPV and (f) MEH-PPV–MEH-PPV.

Distances between the centres of mass of rings were calculated by radial distribution functions (RDF), from the first peaks position. Table 2 shows the average distance between the rings is ≈1.0 nm. A wide variation found for the position of the first peaks is a characteristic of non-crystalline materials, see Fig. S2 in the ESI.† It is recognised that the distance between the aromatic ring centroids for face-to-face and edge-to-face interactions are smaller than 0.40 nm and 0.50 nm in organic materials, respectively.^56–58^ Therefore, face-to-face π–π stacking interactions are less feasible than herringbone-like (edge-to-face) packing as illustrated in the wall-eye stereogram in Fig. 7(a)–(c). The difference of solvent polarity influenced mainly the interactions between FO–FO rings, as shown in Fig. 7(d). This can be explained by the cohesional entanglements induced by THF, in other words, poor solvents induce the nearest interactions between the FO rings.

**Table 2:** Average distances of centre of mass between rings, calculated from the RDF

Ring interactions	*r̄*_toluene_ (nm)	*r̄*_THF_ (nm)
FO–FO	0.93	0.88
FO–MEH-PPV	1.01	1.00
MEH-PPV–MEH-PPV	0.95	0.95

## 4 Conclusions

In this work, we have compared the structural effects in the chains of the bulk of PFO–MEH-PPV films built from THF and toluene, using atomistic molecular dynamics simulations. The solvent molecules were systematically and gradually removed from these systems until they were completely excluded. The formation of distinct intermolecular arrangements are different between the films. In a good solvent such as toluene, the intermolecular distances between the chains of the film are smaller than those built from THF. The backbone chains in the former film showed a significant occurrence of topological-like entanglements over those in the latter film, and the occurrence of π–π-stacking interactions becomes more difficult. On the other hand, the THF induced local parallel alignments to another adjacent segment promote cohesional entanglements, and the rising of some π–π-stacking interactions. Thus, the final molecular architecture of the films was induced by the nature of solvent chosen during the film processing. According to the results, the MD simulations are sensitive tools to provide additional insight and analysis on the structural conformations of chains in the bulk of polymer films originating from different solvents.

## Conflicts of interest

There are no conflicts to declare.

## Supplementary Material

RA-010-D0RA02058C-s001
