# Supplementary material for: Solvent influence on molecular interactions in the bulk of fluorene copolymer films
Source: RSC Adv. 2020 Jun 1;10(35):20772–7. doi: 10.1039/d0ra02058c (PMC9054281; doi:10.1039/d0ra02058c)
Supplement: RA-010-D0RA02058C-s001 [file RA-010-D0RA02058C-s001.pdf]

Electronic Supplementary Material (ESI) for RSC Advances.
This journal is © The Royal Society of Chemistry 2020

# Electronic Supplementary Information

## Solvent influence on molecular interactions in the bulk of fluorene copolymer films

Karina da Silva Dias,[a,‡] Ranylson Marcello Leal Savedra,[a,b] Carlos Eduardo Tavares de Magalhães[a] and Melissa Fabíola Siqueira[a,b,*]

[a]MolSMat - Molecular Simulation of Materials/Laboratory of Computational Simulation (LabSimCo), Department of Physics, Federal University of Ouro Preto, 35400-000, Ouro Preto, MG, Brazil.

[b]Laboratory of Polymers and Electronic Properties of Materials (LAPPEM), Department of Physics, Federal University of Ouro Preto, Ouro Preto, MG, Brazil.

[‡]Present address: Department of Physics, Federal University of Santa Catarina, Florianópolis, SC, Brazil.

[*]To whom correspondence should be addressed.

M. F. Siqueira
E-mail: melissa@ufop.edu.br

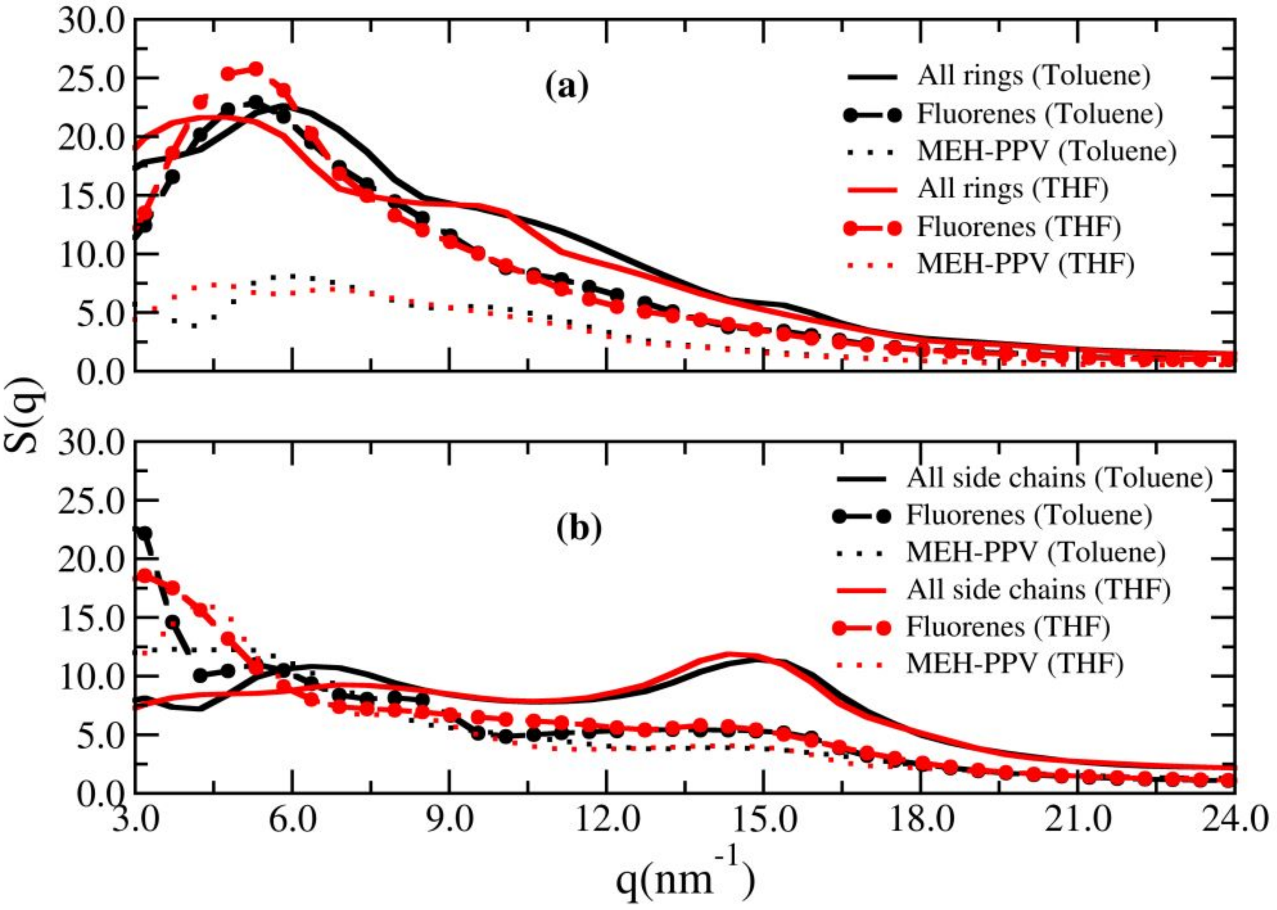


Fig. S1 Structure factors contributions, in different solvents, calculated for (a) rings and (b) sidechains.

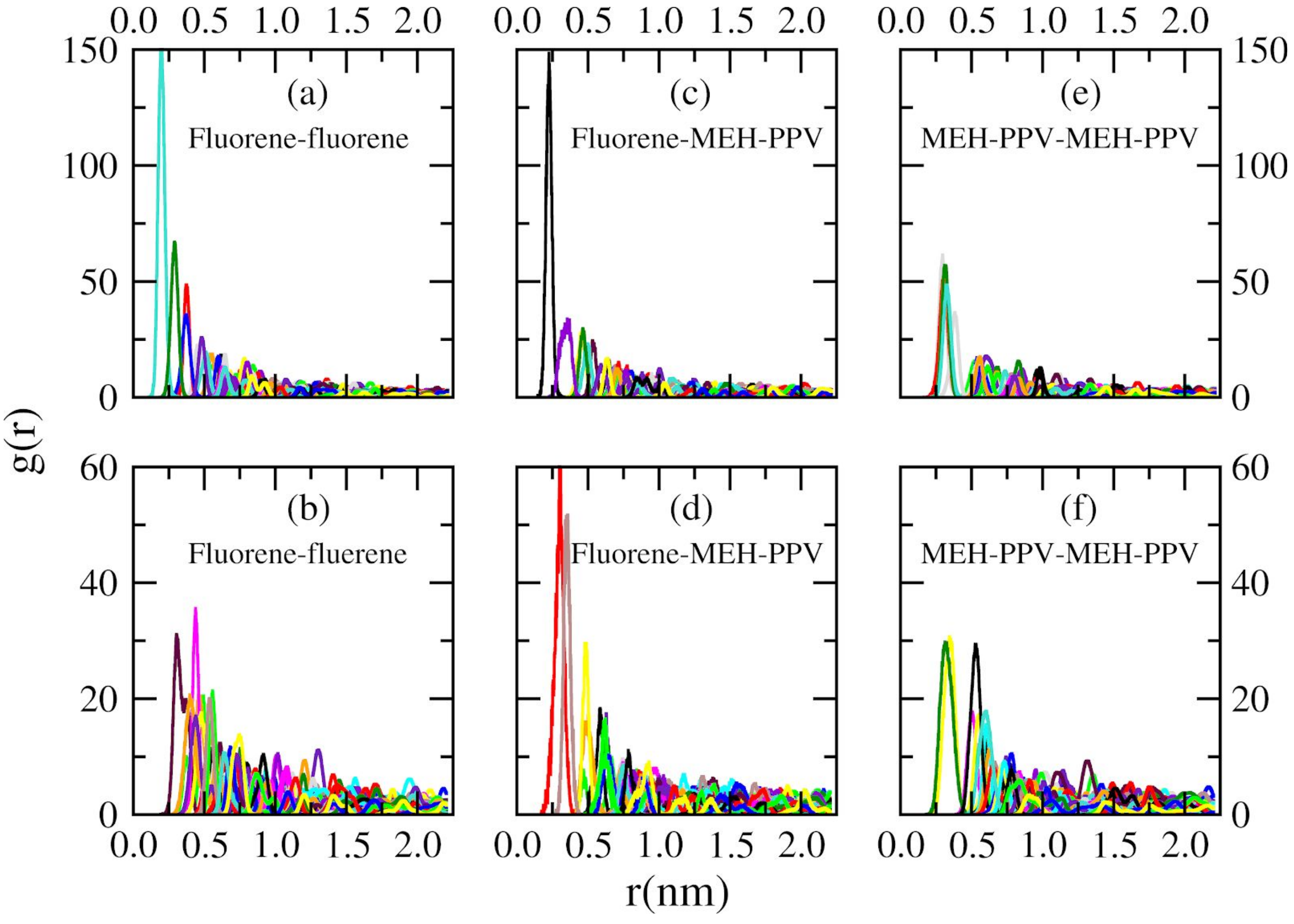


Fig. S2 Radial distribution functions (RDF) calculated for the interaction between rings. Depictions of interactions between FO-FO (a and b), FO-MEH-PPV (c and d) and MEH-PPV-MEH-PPV (e and f). On top are shown RDFs calculated for the film built from THF and on the bottom from toluene.

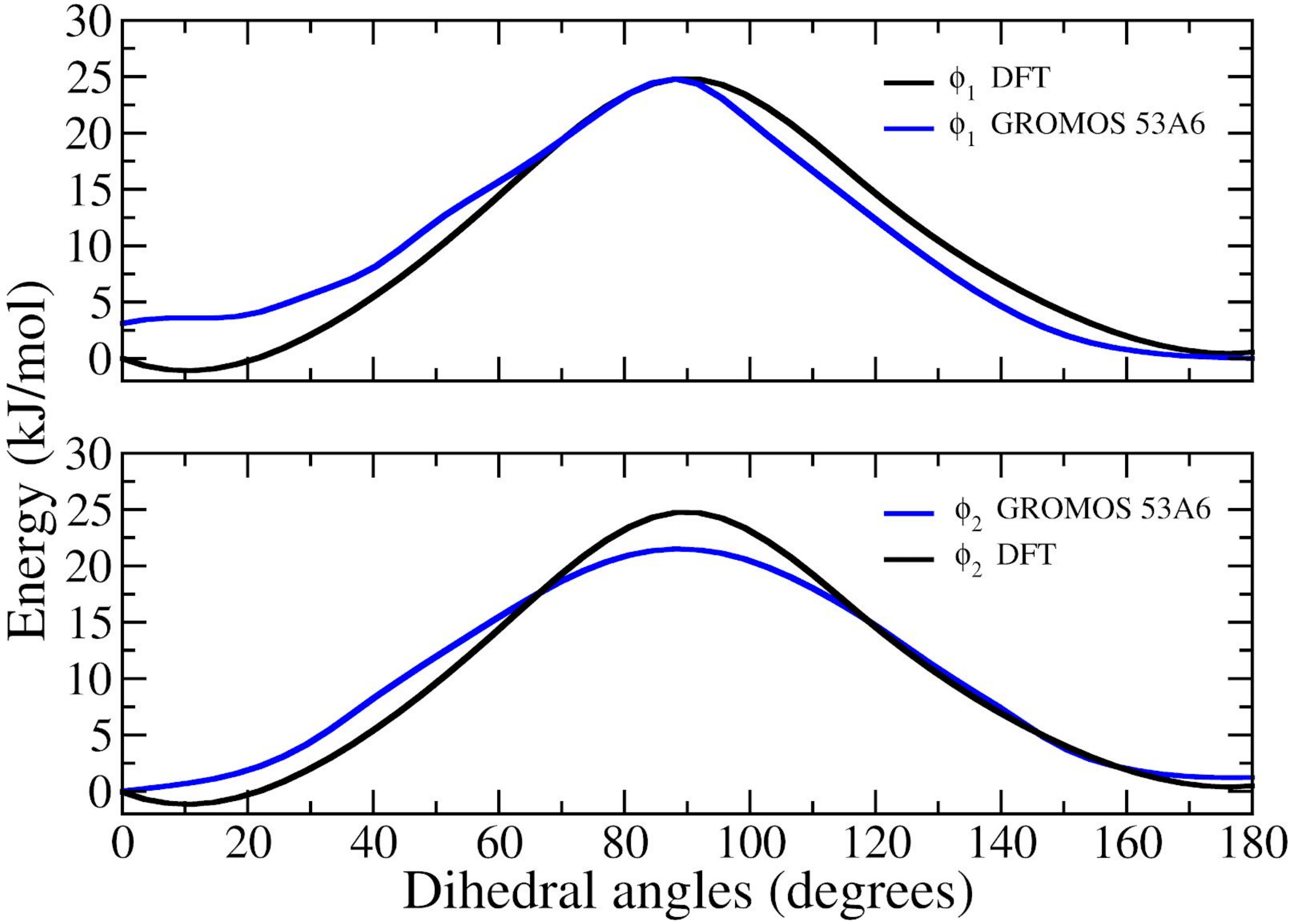


Fig. S3 Potential energies as a function of dihedral angles, calculated using B3LYP/6-31G** and the force field GROMOS 53A6, for the C-C single bonds evaluated in Figure 6 (a) and (c). The top panel shows the potential energy calculated for the fluorene–vinyl (ϕ1), and bottom panel contains the curves for the MEH-PPV–vinyl (ϕ2).
